# Effects of Pre-Germination Assisted with Ultrasound on the Total Flavonoids and Water Absorption Process and Structural Characterization of Starch in Tartary Buckwheat Seeds

**DOI:** 10.3390/foods15213920

**Published:** 2026-11-02

**Authors:** Ling Zhang, Zhe Cheng, Ting Zhang, Yunlong Li

**Affiliations:** Shanxi Institute for Functional Food, Shanxi Agricultural University, Taiyuan 030031, China

**Keywords:** Tartary buckwheat seeds, pre-germination assisted with ultrasound, distribution of moisture, flavonoids, the structural characteristics of starch

## Abstract

Tartary buckwheat is a characteristic minor grain crop rich in flavonoids, while pre-germination is an effective strategy to improve its nutritional quality. Ultrasound, as a green physical pretreatment technology, can modulate seed germination and starch structural properties. However, the multi-scale structural evolution of Tartary buckwheat starch and nutrient accumulation during ultrasound-coupled pre-germination remains insufficiently understood. This study explored the influences of ultrasound-assisted pre-germination on the nutritional components and total flavonoid content (TFC) of Tartary buckwheat seeds. Meanwhile, the variations in water absorption behavior, internal water distribution, and multi-scale starch structural characteristics were systematically analyzed under different ultrasonic treatment durations. The results indicated that soaking prior to ultrasound treatment better promotes water absorption and TFC of pre-germination Tartary buckwheat. Under the optimal processing conditions of 3 h soaking time, 60 min ultrasound treatment and 20 h pre-germination time, the contents of protein, fat, total starch, amylose and TFC in the Tartary buckwheat seeds peaked. However, there were no significant differences in protein and fat contents between the 50 min and 60 min ultrasound treatments, nor were significant differences observed in amylose and resistant starch contents between the 60 min and 70 min ultrasound treatments. Furthermore, ultrasonic treatment for 40–50 min increased the fraction of small starch granules. Upon extending sonication to 50–60 min, intermolecular hydrogen bonding, relative crystallinity (RC) and short-range ordered structure were all enhanced. Compared with 60 min ultrasound treatment, prolonged sonication (70–80 min) promoted the growth of amorphous regions and the formation of large granule agglomerates. Meanwhile, the short-range ordered structure decreased, and the crystalline framework was partially disrupted; nevertheless, X-ray diffraction (XRD) and Fourier transform infrared (FTIR) results demonstrated that ultrasound-assisted pre-germination did not alter the crystalline polymorph of Tartary buckwheat starch. This finding provides a theoretical basis for the green processing and quality improvement of Tartary buckwheat.

## 1. Introduction

Tartary buckwheat (*Fagopyrum tataricum* (L.) Gaertn.) is well recognized for its outstanding nutritional value. A variety of bioactive substances have been identified in Tartary buckwheat seed, including flavonoids, phenolic acids, resistant starch and resorcinols. Among these, flavonoids are one of the most abundant and crucial bioactive substances [1]. Tartary buckwheat seeds are of great research value and application potential in the food and pharmaceutical industries. Furthermore, extensive studies have demonstrated that flavonoids in Tartary buckwheat exhibit various beneficial effects, including hypoglycemic, hypolipidemic, and cholesterol-lowering activities, as well as free radical scavenging, antioxidant, anti-tumor, and anti-aging properties [2,3]. Furthermore, Tartary buckwheat starch is rich in resistant starch, which cannot be hydrolyzed by digestive enzymes in the small intestine of healthy humans. Resistant starch passes intact into the large intestine, where it is fermented and utilized by gut microbiota. Possessing physiological functions analogous to dietary fiber [4], it exerts prebiotic effects, such as lowering total cholesterol (TC) and triglycerides (TG), alleviating the risk of colon cancer and other intestinal disorders, and maintaining intestinal homeostasis [5]. Tartary buckwheat is frequently processed into noodles [6,7], rice [8], tea [9,10], vinegar [11] and liquor [12], which have gained increasing popularity among consumers. However, the processing of these products often results in significant losses of functional components, particularly flavonoids [13]. Therefore, enhancing seed quality and functionality through advanced processing techniques has emerged as a key research focus in the current Tartary buckwheat industry.

Germination is an effective strategy for improving plant tissue structure and enhancing nutritional quality [14,15,16]. Clinical and epidemiological studies have demonstrated that regular consumption of sprouted whole grains helps reduce cholesterol levels, alleviate diabetes risk, inhibit tumorigenesis and regulate the gut microbiota [17,18,19,20,21,22]. Furthermore, various techniques have been applied to facilitate seed germination, and these methods have been proven to improve the germination efficiency, including salt stress [23], anaerobic stress [24], sugar treatment [25], acid solution treatment [26], enzyme treatment [27] and ultrasonic treatment [28]. Among these methods, ultrasound, as a non-invasive and facile processing technology, exerts a positive effect on promoting intracellular mass transfer, stimulating enzyme activity and enhancing cellular permeability and metabolism [29,30]. In recent years, ultrasonic treatment has been applied by researchers to treat cereal seeds during germination, leading to a remarkable accumulation of bioactive substances [31]. During the germination of Tartary buckwheat seeds, functional components including flavonoids accumulate, thereby enhancing the biological activity of the seeds [32]. However, newly formed sprouts are prone to detachment during subsequent processing, which inevitably causes the loss of functional components, particularly flavonoids. Accordingly, precisely regulating seeds physiological status to maintain the pre-germination stage (prior to sprout emergence) is conducive to minimizing the loss of flavonoids and other active substances in Tartary buckwheat products. To date, the effects of ultrasound-assisted pre-germination on the nutritional quality and flavonoids profiles of Tartary buckwheat seeds remain scarce. In this study, the ultrasound-assisted germination conditions for Tartary buckwheat were optimized with flavonoid content as the evaluation index. Additionally, the influences of the optimized treatment on nutritional and functional components, moisture dynamics, as well as starch structural characteristics were systematically investigated.

## 2. Materials and Methods

### 2.1. Tartary Buckwheat Seeds Samples and Pre-Germination Treatment

The selected Tartary buckwheat seeds variety in the experiment was JinQiao 2 purchased from the market (Shanxi, China, 2024, Stored at 4 °C). The initial moisture content of the rough Tartary buckwheat seeds was approximately 11–12% (w.b.). The initial germination rate was 0, and the total flavonoid content was approximately 20 mg/g.

The pre-germinated Tartary buckwheat seeds (experimental groups that undergo sequential soaking, ultrasonic treatment and re-soaking before radicle emergence) were dried in a hot-air oven at 45 °C until the moisture content reached approximately 10%. Following drying, the seeds were utilized for determinations of water absorption and distribution. After dehulling, grinding and sieving through a 100-mesh screen, the obtained powder was used for analyses of nutrient and flavonoid contents, as well as for starch extraction and subsequent structural characterization. The raw control seeds were subjected to the same drying, dehulling and milling treatments.

### 2.2. Chemicals and Reagent

Resistant Starch Assay Kits were purchased from Megazyme Co., Ltd., Bray, Ireland. Standard solution of potato amylose was purchased from MACKLIN Co., Ltd., Shanghai, China. All the chemicals were analytical grade.

### 2.3. Experimental Design

#### 2.3.1. Magnetic Resonance Imaging (MRI) Analysis of Internal Water Distribution in Tartary Buckwheat Seeds and Experimental Design of Ultrasound Assistance at Different Stages

To elucidate the internal water uptake process of Tartary buckwheat seeds, MRI was employed to track water distribution and migration during hydration, so as to optimize the soaking time. Meanwhile, to verify whether ultrasound technology can effectively facilitate the pre-germination of Tartary buckwheat seeds, and determine the optimal ultrasound application stage (i.e., ultrasound-assisted treatment before soaking or during soaking), a series of experiments were designed with a total soaking duration of 7 h.

Y1: Dry Tartary buckwheat seeds were soaked directly for 7 h.
Y2: Dry Tartary buckwheat seeds were subjected to 1 h ultrasound treatment, followed by 7 h of soaking.
Y3: For a total soaking duration of 7 h, dry Tartary buckwheat seeds were first soaked for 1 h. After draining and blotting surface moisture from the seeds, the seeds were treated with ultrasound for 1 h and then soaked for a further 6 h.

#### 2.3.2. Single-Factor Experiment for Optimization of Ultrasound-Assisted Pre-Germination Conditions of Tartary Buckwheat Seeds

##### Soaking Time

Tartary buckwheat seeds (100 g) were soaked in 300 mL water for 1 h, 2 h, 3 h, 4 h and 5 h. After soaking, the seeds were drained of surface water, transferred into sealed bags, and then treated by 60 min of ultrasound treatment using an intelligent ultrasonic cleaner (RS-180E, Shanghai Ruisheng Instrument & Meter Co., Ltd., Shanghai, China). This instrument is equipped with adjustable ultrasonic power, timing function and temperature control function. (40 kHz, 180 w, 60 min, 25 °C). Subsequently, the seeds were subjected to 20 h pre-germination (the seeds were kept from sprouting) at a solid-to-liquid ratio of 1:3 (seed-to-water, *w*/*v*), and 25 °C in an incubator. After pre-germination, the content of flavonoid was measured to screen the optimal soaking time.

##### Ultrasound Time

Tartary buckwheat seeds (100 g) were soaked in 300 mL water for 3 h (optimal soaking time), and then subjected to ultrasound treatment for 40 min, 50 min, 60 min, 70 min and 80 min. Subsequently, the seeds underwent 20 h pre-germination at 25 °C. After pre-germination, the content of flavonoid was measured to screen the optimal ultrasound duration.

##### Pre-Germination Time

Tartary buckwheat seeds (100 g) were soaked in 300 mL water for 3 h, followed by 60 min of ultrasound treatment. The seeds were subsequently pre-germinated for 16 h, 18 h, 20 h, 22 h and 24 h at 25 °C. After pre-germination, the content of flavonoid was measured to screen the optimal pre-germination duration.

#### 2.3.3. Response Surface Experiment for Optimization of Ultrasound-Assisted Pre-Germination Conditions of Tartary Buckwheat Seeds

On the basis of single-factor experiments, a three-factor, three-level response surface methodology (RSM) was adopted for optimization. Soaking time (A), ultrasound treatment duration (B) and pre-germination duration (C) were set as independent variables, while total flavonoid content served as the response variable. The corresponding factor levels are shown in Table 1.

### 2.4. Determination of Nutritional Components

Moisture (GB 5009.3-2016 [33]), protein (GB 5009.5-2016 [34], with a nitrogen-to-protein conversion factor of 6.25), fat (GB 5009.6-2016 [35]), total starch (GB 5009.9-2016 [36], acid hydrolysis method) and amylose contents (GB/T 15683-2025 [37]) were determined according to the Chinese national standards (GB). The contents of resistant starch were analyzed, following the method described in the kit instructions. Exactly 100 mg of sample was weighed into a test tube, and 4.0 mL of pancreatic α-amylase solution (10 mg/mL) was added to each tube. After incubation at 37 °C for 16 h, 4.0 mL of ethanol (99%, *v*/*v*) was added to terminate the reaction. The mixture was centrifuged at 1500 *g* for 10 min, and the pellet containing resistant starch (RS) was collected and weighed. All the above components were measured three times using the prepared samples and expressed on a dry-weight basis.

### 2.5. Determination of Flavonoid

The flavonoid content of Tartary buckwheat seeds was measured according to the method described by Liu et al. [1]. A sample of 0.2 g was weighed and extracted with 50 mL of 70% methanol at 70 °C for 3 h to obtain the sample extract. Exactly 1 mL of the sample solution was transferred into a 10 mL stoppered colorimetric tube. Subsequently, 2 mL of 0.1 mol/L AlCl_3_ solution and 3 mL of 1 mol/L CH_3_COOK solution were added sequentially, and the mixture was diluted to 10 mL with 70% methanol. A sample blank was prepared in parallel following the same procedure. After thorough shaking, the solution was allowed to stand for 30 min at dark, and its absorbance was determined at 420 nm using an Ultraviolet-Visible Spectrophotometer (T500F, Beijing Puxi General Instrument Co., Ltd., Beijing, China).

Preparation of the standard curve. Exactly 0, 0.5, 1, 2, 3 and 4 mL of 0.05 mg/mL rutin standard solution were transferred into separate 10 mL stoppered colorimetric tubes. Each tube was subjected to the identical color-development procedure described above, and the absorbance was recorded. The standard curve was plotted with rutin concentration on the x-axis and the corresponding absorbance on the y-axis. The regression equation was y = 3.2653x + 0.0059, with a coefficient of determination R^2^ = 0.9998. The measured absorbance of samples was substituted into this regression equation to calculate the total flavonoid content.

### 2.6. Determination of Water Distribution in the Pre-Germinated Seeds

The water distribution in the seeds was determined using (MRI, NM120-060 h-I, Shanghai Niumag Electronic Technology Co., Ltd., Shanghai, China) according to the method described by Zou et al. [38]. Samples with different ultrasound treatments and pre-germination were soaked for 0 h, 2 h and 4 h, respectively. After surface moisture was blotted dry, the samples were placed in the center of the MRI tube coil. A 25 mm probe was adopted, and the free induction decay (FID) sequence was used to calibrate the center frequency and radio frequency. Subsequently, the spin-spin relaxation time (T2) was measured using the CPMG pulse sequence. The CPMG sequence was set as follows: main frequency (SF) = 21 MHz, offset frequency = 198,842.5 Hz, repetition time (TW) = 1500 ms, 90° pulse width = 6.00 μs, 180° pulse width = 10.24 μs, number of scans = 8, and preamplifier gain = 0. The data were analyzed using NiumagT-Invertfit 4.08 software. All peak areas were normalized by sample mass.

### 2.7. Determination of Water Absorption Rate

The water absorption rate of the seeds was measured according to the method described by Lian et al. [39]. Accurately, 5.0 g of pre-germinated Tartary buckwheat seeds from different treatments were weighed into beakers (precision: 0.001 g). Subsequently, 25 mL of distilled water was added, and the beakers were incubated in a constant-temperature water bath at 25 °C. Samples were collected at 1 h intervals, and surface moisture was gently blotted with cheesecloth prior to weighing. The water absorption rate (%) was calculated according to the following equation:

Water absorption rate (%) = [(m_2_ − m_1_)/m_1_] × 100%

where m_1_ is the sample mass before water absorption (g), and m_2_ is the sample mass after water absorption (g).

### 2.8. Starch Extraction Process

Pre-germinated Tartary buckwheat flour samples were blended with an appropriate amount of distilled water and homogenized into a slurry. The slurry was sieved through a 100-mesh screen, and the filtrate was allowed to stand for 2 h. After the supernatant was discarded, the precipitate was resuspended in deionized water and centrifuged at 4000 r/min for 15 min. The washing and centrifugation procedures were repeated several times to purify the starch precipitate. Finally, the purified precipitate was freeze-dried, pulverized, and sieved again through a 100-mesh screen. The obtained powder was subsequently used for scanning electron microscopy (SEM) observation, iodine absorption measurement, crystalline structure analysis and Fourier transform infrared (FTIR) spectroscopy characterization.

### 2.9. Determination of Starch Microstructure

The granular morphology of Tartary buckwheat starch was characterized using a scanning electron microscope (SEM, SM-7900F Thermal Field Emission, JEOL Ltd., Tokyo, Japan) according to the method described by Fu et al. [40]. Conductive double-sided adhesive tape was affixed to an aluminum stub, and a small quantity of dried starch granules was evenly mounted on the tape surface. All samples were sputter-coated with a thin gold layer before observation. Starch granule micrographs were acquired at a magnification of 1500× to analyze the variations changes in morphological characteristics.

### 2.10. Determination of Iodine Absorption Property

Iodine absorption properties were analyzed as described by Jiang et al. [41]. A 20 mg starch sample was weighed, moistened with 0.5 mL absolute ethanol, and dissolved in 1 mL of 2 mol/L potassium hydroxide (KOH). The pH was adjusted to 6.0–7.0, and the resulting solution was quantitatively transferred into a 50 mL volumetric flask and diluted to the mark. An aliquot (2.5 mL) of the above solution was mixed with 0.2 mL iodine reagent, and the mixture was diluted to a final volume of 10 mL. The absorbance spectrum of the prepared solution was scanned over the wavelength range of 500–800 nm. The degree of polymerization (*DP*) was calculated using the following equation:

$$DP=\frac{0.01025}{\frac{1}{\lambda_{max}}-0.001558}$$

where *λ*_max_ (nm) represents the maximum absorption wavelength obtained from UV–vis spectroscopy.

### 2.11. Determination of Starch Crystalline Structure

XRD analysis of the starch samples was performed on a D8 ADVANCE A25 X-ray powder diffractometer (Beijing Aikexirui Technology Co., Ltd., Beijing, China) following the method of Chen et al. [42]. An appropriate quantity of starch sample was evenly loaded onto the sample holder. XRD patterns were acquired with monochromatic Cu-Kα radiation. The scanning conditions were set as follows: 2θ range from 4° to 60°, step size of 0.02°, scanning rate of 4°/min and continuous-scan mode. The relative crystallinity was calculated and analyzed using MDI Jade 6.0 software.

### 2.12. Determination of Starch FTIR Spectroscopy

Starch FTIR spectra were recorded using a Fourier Transform Infrared Spectrometer (Nicolet iS5, Thermo Fisher Scientific, Waltham, MA, USA) according to the method described by Li et al. [43]. An amount of 1 mg of starch sample and 100 mg of potassium bromide (KBr) were accurately weighed, homogenized and pressed into a transparent thin pellet. The spectrometer was set to a scanning range of 4000 to 400 cm^−1^ with a resolution of 4 cm^−1^, and 64 scans were accumulated for each spectrum. Additionally, the spectra in the range of 1200 to 800 cm^−1^ were subjected to baseline and subsequent deconvolution analysis. The spectra were baseline-corrected and normalized to a range of 0–100 using Origin 2018 software. The ratios DO (1047/1022 cm^−1^) and DD (1022/995 cm^−1^) were calculated using PeakFit 4.12 software to evaluate the short-range molecular order of starch.

### 2.13. Data Processing

SPSS 26.0 software was used for data analysis, and Origin 2018 was applied for data plotting. The response surface data were analyzed by Design-Expert 8.0.6.

## 3. Results and Discussion

### 3.1. Process Optimization Experiment Analysis

#### 3.1.1. Effects of Ultrasound-Assisted Pre-Germination on the Moisture of Tartary Buckwheat Seeds

MRI enables the visualization of the spatial distribution and dynamic migration of water in the samples. In the acquired MRI images, color gradients correspond to proton density: blue represents low density, whereas yellow and red indicate high density, and yellow-green specifically reflects the presence of water. As presented in Table 2, the MRI images of Y1, Y2 and Y3 groups displayed low clarity after 1 h of soaking, suggesting limited water uptake, and Y1 exhibited the lowest water absorption capacity. With extended soaking time, the images resolution was markedly improved, accompanied by the expansion of yellow-green regions and a gradual rise in moisture content. Both Y1 and Y2 reached full imaging saturation at 7 h; however, Y2 presented a higher yellow-green density, indicating that water absorption had reached saturation. In comparison, Y3 achieved complete imaging at just 5 h, featured by distinct boundaries and elevated yellow-green density, which demonstrated that it reached water absorption saturation earlier than the other samples.

During the entire soaking process, the ultrasound-assisted treatments (Y2 and Y3) exhibited larger yellow-green regions and clearer structural contours in MRI images compared to the non-ultrasound treatment (Y1). Notably, at 5 h of soaking, the imaging boundaries of Y3 were markedly sharper and more intact than those of Y1 and Y2. Meanwhile, Y3 presented the highest yellow-green density, indicating a deeper degree of water infiltration into the interior of the Tartary buckwheat seeds. These results confirmed that ultrasound-assisted pre-germination accelerates water uptake in Tartary buckwheat seeds, and the sequential treatment of soaking followed by ultrasound prior to pre-germination achieves the strongest promoting effect.

#### 3.1.2. Effect of Ultrasound-Assisted Pre-Germination on TFC of Tartary Buckwheat Seeds

Based on the water migration characteristics via MRI, further experiments were designed with TFC as the evaluation index to further elucidate the effects of ultrasound-assisted pre-germination. The experimental treatments were set as follows: S1, continuous soaking for 10 h; S2, 1 h of ultrasound followed by 10 h of continuous soaking; S3, initial soaking for 1 h, surface moisture removal, 1 h ultrasound treatment, and subsequent re-soaking for 9 h; S4, initial soaking for 4 h, surface moisture removal, 1 h ultrasound treatment, and subsequent re-soaking for 6 h; S5, initial soaking for 7 h, surface moisture removal, 1 h ultrasound treatment, and subsequent re-soaking for 3 h.

**Figure 1:**
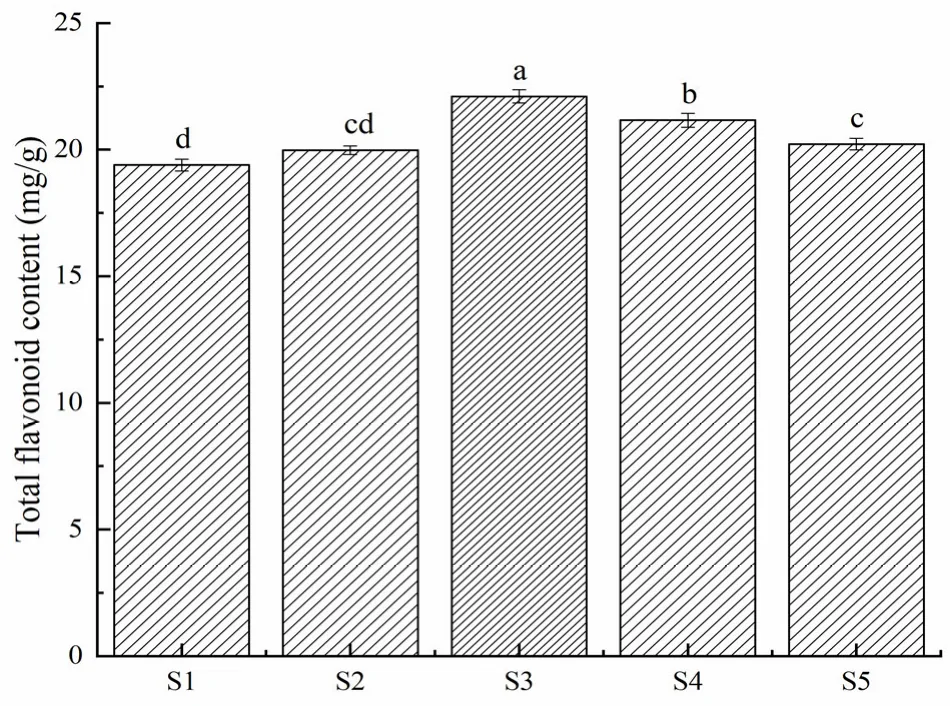
Pre-experiment of ultrasound-assisted germination to increase TFC. S1: soaking 10 h; S2: ultrasound 1 h + soaking 10 h; S3: soaking 1 h + ultrasound 1 h + soaking 9 h; S4: soaking 4 h + ultrasound 1 h + soaking 6 h; S5: soaking 7 h + ultrasound 1 h + soaking 3 h. Different letters show significant variations (*p* < 0.05).

As presented in Figure 1, the TFC of the ultrasound-assisted pre-germination sample without prior soaking (S2) was slightly higher than that of the non-ultrasound control (S1). Moreover, the TFC of ultrasound-assisted pre-germination seeds with prior soaking (S3, S4 and S5) was significantly higher than those of both S1 and S2. The sequential treatment of preliminary soaking combined with subsequent ultrasound-assisted pre-germination achieved a significantly enhanced effect. Collectively, these findings indicated that pre-hydration to an appropriate moisture state prior to ultrasound treatment is more conducive to the improvement of TFC in Tartary buckwheat seeds.

For S1 (soaking alone), the intact seed coat retarded imbibition, key flavonoid-synthesizing enzymes exhibited low activities, and TFC was minimal [31]. Ultrasonic cavitation disrupted the compact Tartary buckwheat seed coat to accelerate water penetration, upregulating flavonoid-synthetic enzymes and stimulating flavonoid biosynthesis. Short pre-soaking primed seeds from dormancy to metabolic activation, enabling ultrasound to effectively induce flavonoid synthesis [32]. Premature ultrasound (S2) failed to trigger strong metabolic responses in non-imbibed seeds, leading to marginal flavonoid induction. When ultrasound was delayed (S4, S5), fully hydrated germinating seeds suffered flavonoid leaching; meanwhile, excessive ROS derived from combined ultrasound and high moisture degraded flavonoids and inhibited synthetic enzymes, lowering total flavonoid content [30].

#### 3.1.3. Analysis of Single-Factor Experiments

**Figure 2:**
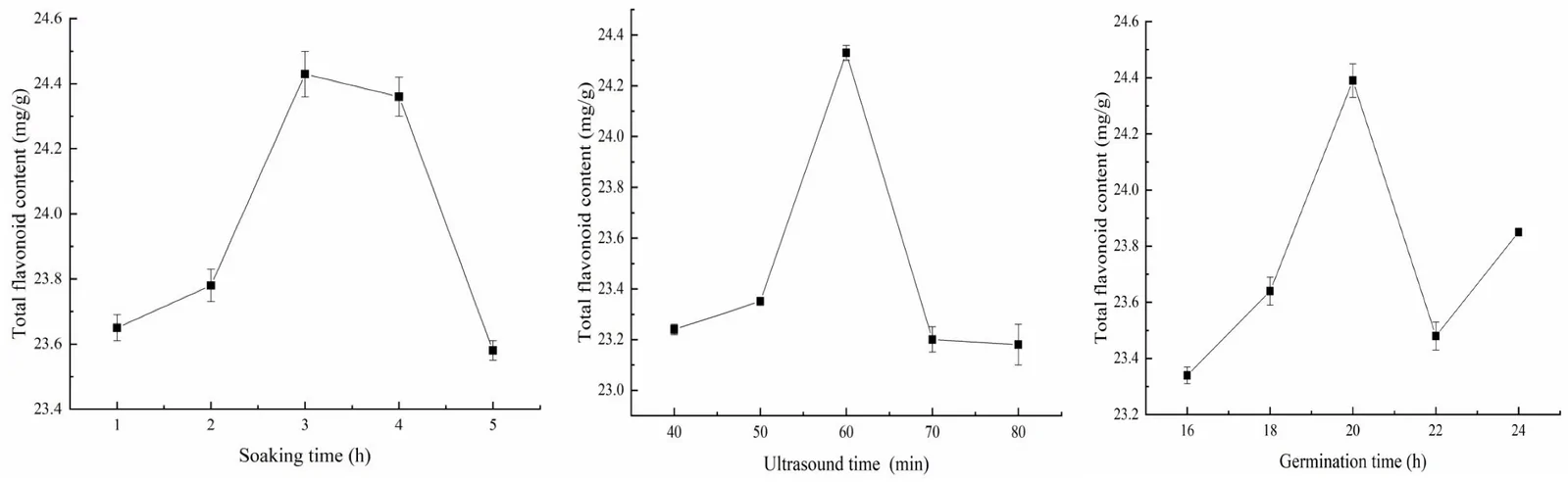
Effect of single-factor test on TFC in Tartary buckwheat seeds.

Preliminary trials were conducted to assess pre-soaking durations ranging from 1 to 5 h. As presented in Figure 2, with the extension of soaking time, the total flavonoid content exhibited an initial increase followed by a decrease and peaked at 3 h. TFC initially increased with ultrasound duration, peaking at 60 min, before declining. Additionally, TFC peaked at 20 h of pre-germination (16–20 h range); however, extending the period to 22–24 h resulted in a reduction in TFC. These results demonstrate that soaking, ultrasonication and pre-germination each have an optimal duration window. Inadequate stimulation fails to fully activate the flavonoid biosynthetic pathway; beyond the critical time threshold, adverse effects including flavonoid leaching, oxidative degradation and precursor depletion predominate, lowering TFC [30,31,32]. This trend agrees with that observed in Figure 1.

#### 3.1.4. Optimization of Ultrasound-Assisted Pre-Germination Process by Response Surface Methodology

Based on the results of response-surface experimental design (Table 3), a mathematical model was constructed and subjected to significance analysis. The experimental data were fitted to a second-order polynomial regression equation, as shown below:

Y = 24.43 − 0.031A − 0.27B − 0.042C − 0.1AB − 0.48AC − 0.27BC − 0.61A^2^ − 0.55B^2^ − 0.35C^2^

Significance tests of the model and regression coefficients are presented in Table 4.

**Figure 3:**
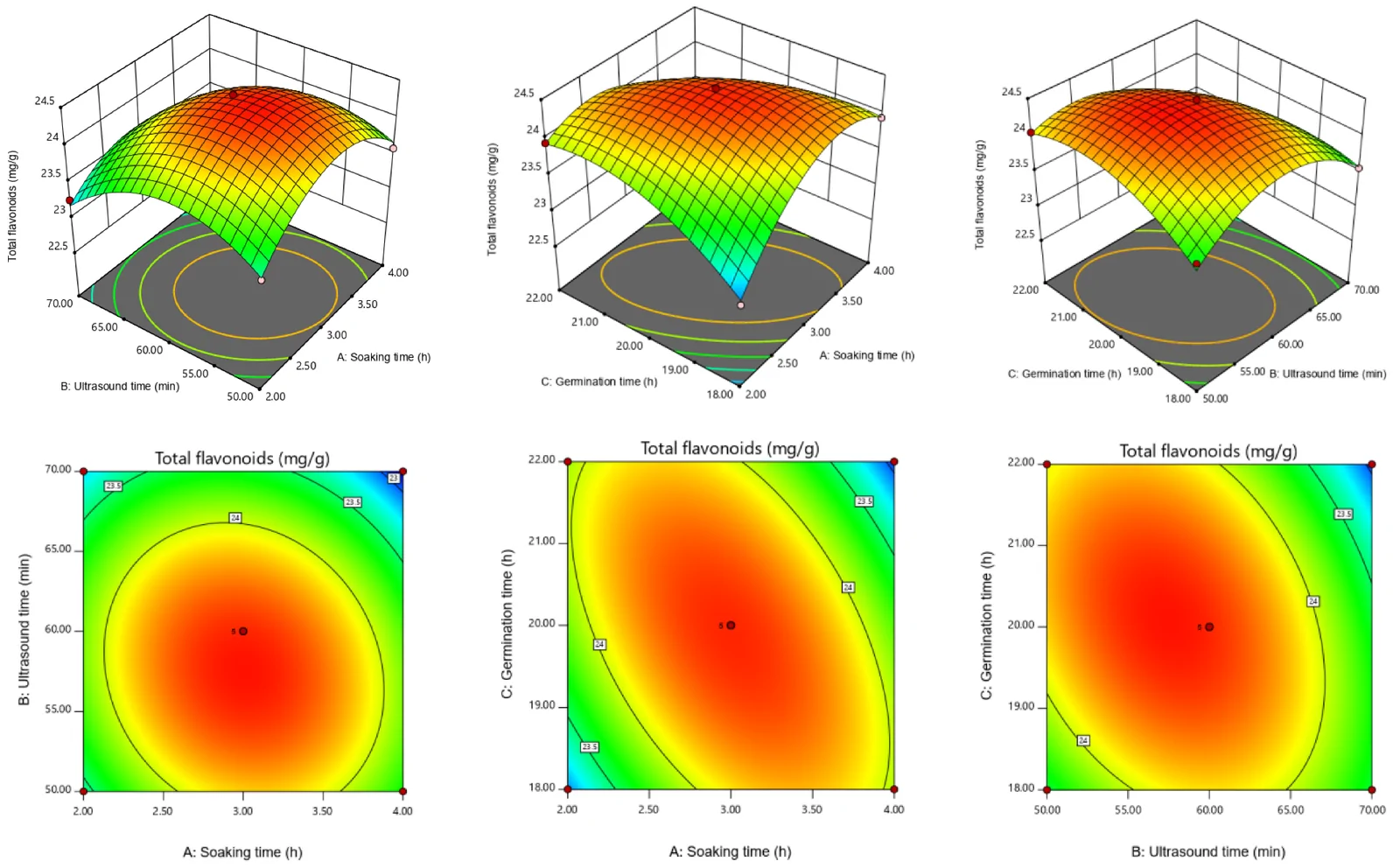
Effects of interaction of various factors on the content of total flavonoids.

The regression model was highly significant (*p* < 0.0001), while the lack-of-fit was non-significant (*p* = 0.0855 > 0.05), suggesting satisfactory model fitness. Accordingly, this model can be applied to predict the response of total flavonoid content to ultrasound-assisted pre-germination treatment. The coefficient of determination R^2^ was 0.9910, the adjusted R^2^ was 0.9795, and the predicted R^2^ was 0.8850. The difference between the adjusted R^2^ and the predicted R^2^ was less than 0.2, further verifying the good consistency between the predicted and experimental values. According to the *F*-values of the regression coefficients, the influence order of the three factors on total flavonoid content was as follows: ultrasound duration > pre-germination duration > soaking time. The effects of individual factors and their interactions on total flavonoid content are illustrated by the 3D response -surface and contour plots presented in Figure 3.

Based on the optimization results from Design-Expert 8.0.6. software, the optimal parameters for ultrasound-assisted pre-germination of Tartary buckwheat seeds were identified: soaking time of 2.99 h, ultrasound treatment duration of 57.41 min, and pre-germination duration of 20.1 h. Under these theoretical conditions, the predicted total flavonoid content was 24.46 mg/g. Considering practical experimental operability, the parameters were rounded to 3 h soaking, 60 min ultrasound treatment, and 20 h pre-germination. The experimentally measured total flavonoid content under the adjusted conditions was 24.45 ± 0.05 mg/g, which showed satisfactory consistency with the predicted value. This result verified the reliability of the regression model and confirmed its practical guiding value.

### 3.2. Effects of Ultrasound Treatment Duration on the Nutritional Composition and Starch Structural Characterization of Pre-Germination Tartary Buckwheat Seeds

#### 3.2.1. Effects of Ultrasound Treatment Duration on the Nutritional and Functional Components of Pre-Germination Tartary Buckwheat Seeds

As shown in Table 5, compared with the untreated raw material, ultrasound-treated pre-germination Tartary buckwheat seeds presented a general increase in protein, fat, resistant starch and TFC. The total starch content peaked at 60 min of ultrasound treatment and then decreased at 70 and 80 min. Amylose content increased significantly at 60 min. Resistant starch content increased slowly with treatment time and reached highest level at 70 min. Zhou et al. [44] found that the contents of total starch, amylose and resistant starch in Tartary buckwheat starch declined steadily with increasing germination duration. It has been reported that moderate ultrasound treatment can significantly raise cassava amylose content by breaking starch chains and releasing bound amylose [45]. Under the tested conditions, shorter ultrasound exposure produced only weak perturbation of seed cells, with limited modification of starch and minor changes in the measured nutritional and bioactive components. By contrast, overly prolonged ultrasound treatment carries the risk of disrupting cell membranes and the ordered packing of starch helices, which may promote the degradation and leaching of storage compounds and bioactive substances [31]. Collectively, 60 min appeared to be the favorable ultrasound duration under the tested conditions, with relatively higher levels of protein, lipid, resistant starch and flavonoids observed, balancing nutritional and functional attributes.

#### 3.2.2. Effects of Ultrasound Treatment Duration on the Water Absorption Rate of Tartary Buckwheat Seeds

**Figure 4:**
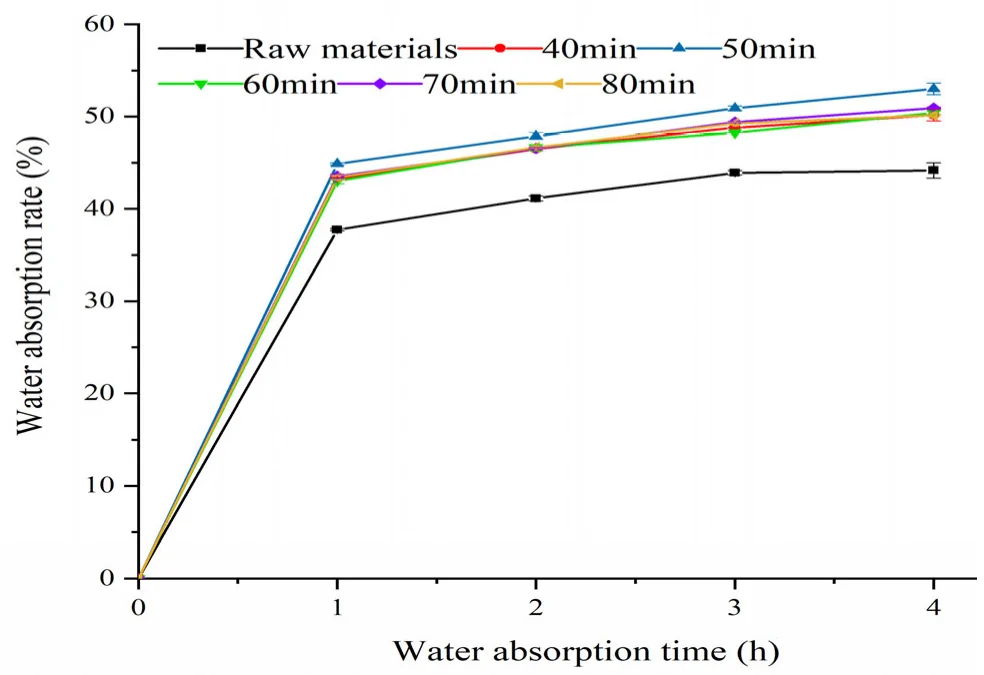
Water absorption rate of Tartary buckwheat seeds with different ultrasonic time.

As shown in Figure 4, the water absorption rate of the seeds subjected to ultrasound-assisted pre-germination was persistently higher than that of the raw seeds during the whole hydration process. Chen et al. [46] suggested that ultrasound-induced cavitation and vibration generate microcracks on the seed pericarp and enhance cell membrane permeability, thereby accelerating water infiltration into seeds. Under the current experimental conditions, these results indicate that ultrasound-assisted pre-germination generally promotes water uptake of Tartary buckwheat seeds. Additional synchronized non-ultrasonic controls will be required in future work to distinguish the individual ultrasonic effect from the influence of pre-germination pretreatment.

#### 3.2.3. Effects of Ultrasound Treatment Duration on the Water Distribution in Tartary Buckwheat Seeds

**Figure 5:**
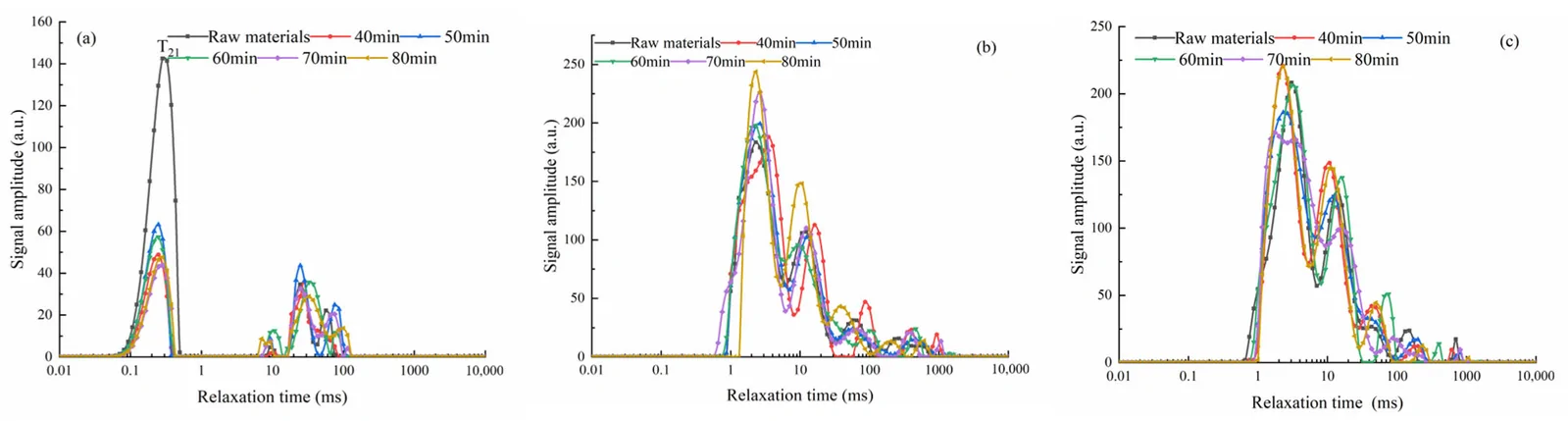
Relaxation time of pre-germination Tartary buckwheat seeds with different ultrasonic time. Note: (**a**) Ultrasound-assisted pre-germinated Tartary buckwheat seeds. (**b**) The ultrasound-assisted pre-germinated Tartary buckwheat seeds were soaked for 2 h. (**c**) The ultrasound-assisted pre-germinated Tartary buckwheat seeds were soaked for 4 h.

The T2 relaxation inversion spectrum covered a range of 0.01–10,000 ms, which was divided into three water populations: T21 (0.1–1 ms) for bound water, T22 (1–100 ms) for immobilized (semi-bound) water, and T23 (100–10,000 ms) for free water. Before hydration, water in raw Tartary buckwheat seeds was dominated by bound water (T21) with a peak area of 954.81, followed by semi-bound water (T22), and free water (T23) was barely detectable (Figure 5a). After 2 h and 4 h of hydration (Figure 5b,c), the bound water content in the raw seeds decreased significantly, with water mainly converting to semi-bound water and the appearance of free water. In comparison, ultrasound-assisted pre-germinated seeds showed a notably smaller T21 peak area (271.61–367.75) prior to hydration, and water was mainly present in the form of bound water and semi-bound water (Figure 5a). After 2 and 4 h of water uptake, bound water in the ultrasound-treated seeds remained at a low level. Notably, the semi-bound water (T22) content in treated seeds was higher than that in raw seeds, accompanied by an obvious free water (T23) peak within the 100–10,000 ms range.

#### 3.2.4. Effects of Ultrasound Treatment Duration on the Morphology of Starch Granules in Tartary Buckwheat Seeds

**Figure 6:**
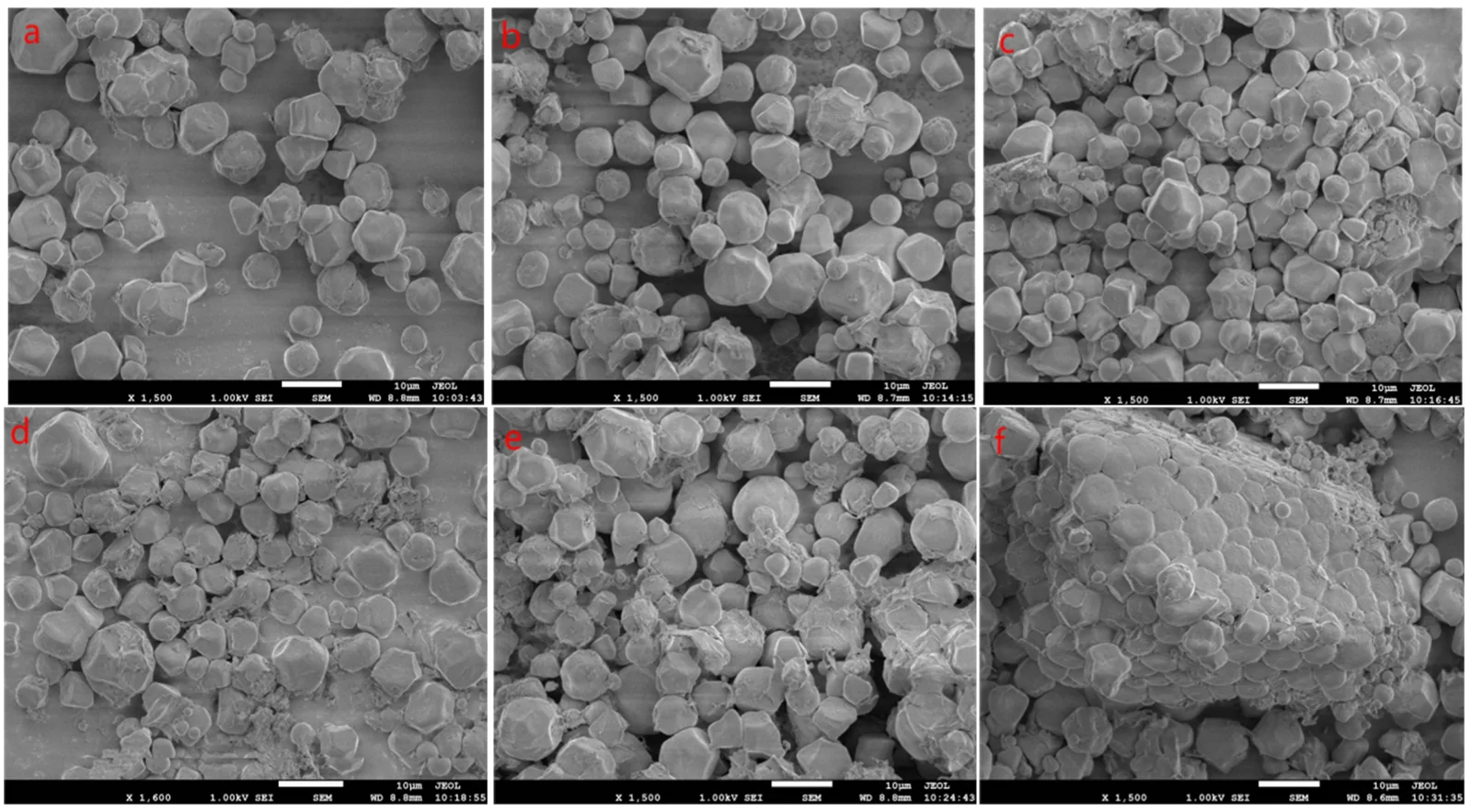
Scanning electron microscope images of Tartary buckwheat starch with different ultrasonic time. Note: The images show scanning electron microscopy (SEM) micrographs of freeze-dried Tartary buckwheat starch (1500×). Panels (**a**–**f**) represent the ultrasound-assisted germinated samples treated for 0 min (raw material), 40 min, 50 min, 60 min, 70 min and 80 min, respectively.

As shown in Figure 6, Starch granules in untreated raw Tartary buckwheat seeds were well dispersed and mostly polyhedral, featuring smooth surfaces, clear boundaries and no obvious structural damage. After 40–50 min of ultrasound-assisted pre-germination treatment, the small starch granules increased, while the overall polyhedral morphology was largely maintained. It can be intuitively observed from Figure 6c that the quantity of small starch granules was abundant at 50 min. Nevertheless, prolonged ultrasound exposure induced obvious granules agglomeration. The most prominent morphological changes to starch granules within Tartary buckwheat seeds were observed after 80 min of sonication, with blurred granule boundaries and intergranular adhesion forming large agglomerates. Wei et al. [47] observed variable surface damage and aggregation in corn and quinoa starch granules after ultrasonic exposure. This trend may arise from amylose assembly through intermolecular hydrogen bonding under the effect of prolonged ultrasonic cavitation, resulting in granule sticking and large aggregate formation [48].

#### 3.2.5. Effects of Ultrasound Treatment Duration on Iodine Absorption Properties of Starch in Pre-Germination Tartary Buckwheat Seeds

**Figure 7:**
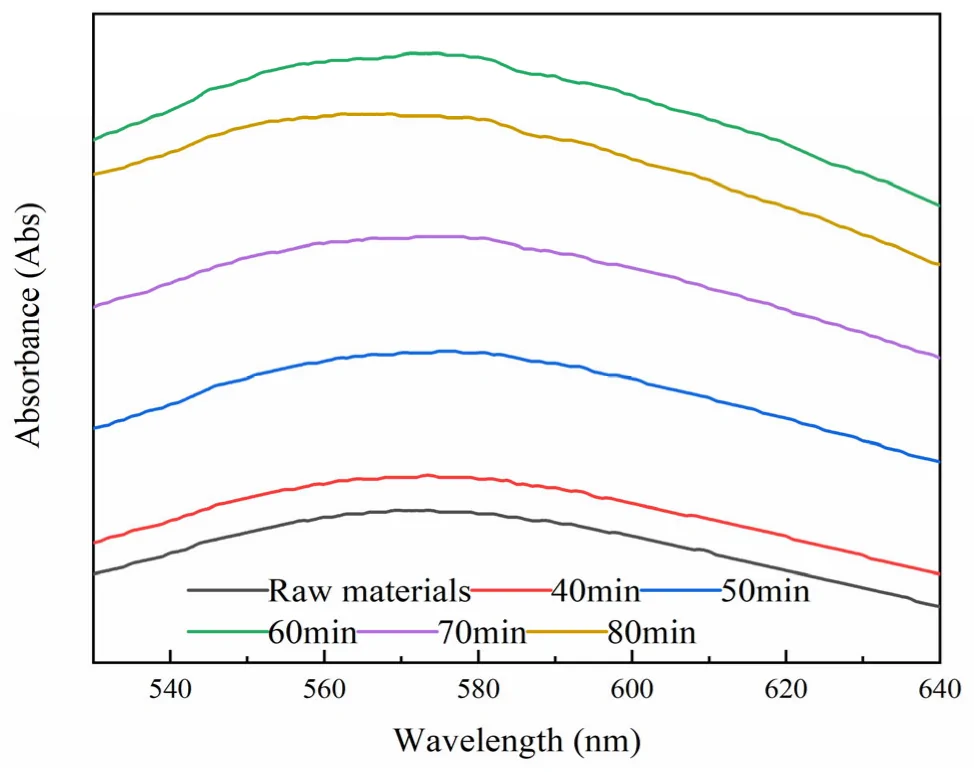
Changes in starch iodine absorption of Tartary buckwheat seeds at different ultrasonic times.

Amylose forms a blue colored complex with iodine, with a maximum absorption peak at 600 to 640 nm. In contrast, amylopectin forms a purplish-red to reddish-brown complex, with its maximum absorption peak at 520–560 nm [49]. As shown in Figure 7, the maximum absorption peak of the raw starch at 571.5 nm, while the peaks of Tartary buckwheat starch treated with different ultrasound durations were at 573.5, 576, 572.5, 575 and 566 nm, respectively. With increasing ultrasound treatment time, no obvious red shift or blue shift was observed for the maximum absorption peak, implying no significant shift in the amylose or amylopectin. The average degree of polymerization (DP) of starch in Tartary buckwheat seeds under different ultrasound-assisted pre-germination treatments was calculated using the Banks equation [50], yielding values of 55.20, 57.55, 54.31, 56.59 and 49.09, respectively. The DP values of samples treated with ultrasound for 40–70 min were all higher than that of raw starch (DP = 53.45). Nevertheless, no clear variation trend was found among the different ultrasound treatment durations. The DP reached its maximum at 50 min of ultrasound treatment. This suggests that ultrasound-assisted pre-germination modifies the iodine-binding properties of Tartary buckwheat starch and exerts an influence on starch chain structure.

#### 3.2.6. Effects of Ultrasound Treatment Duration on the Crystalline Structure of Starch in Pre-Germination Tartary Buckwheat Seeds

**Figure 8:**
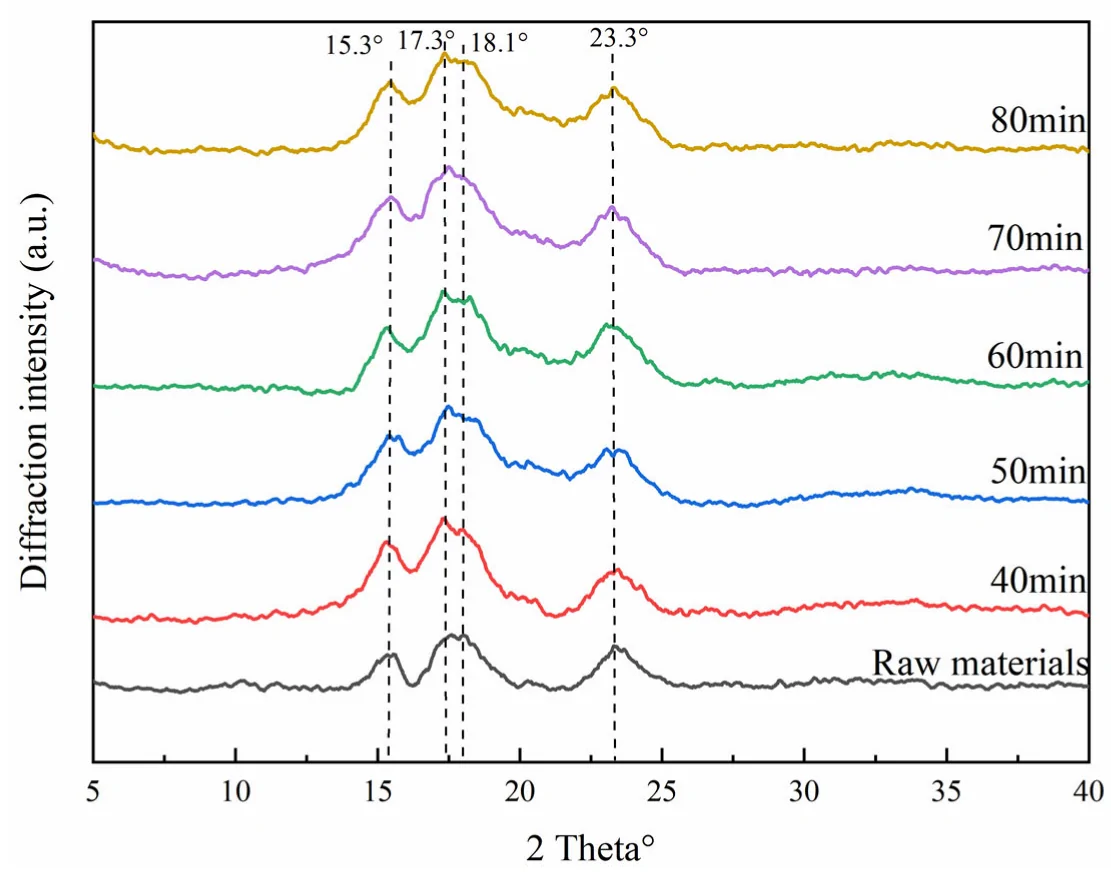
Changes in starch crystal structure of Tartary buckwheat seeds at different ultrasonic time.

XRD analysis generally identifies three typical crystalline patterns of starch: A, B and C. The A-type pattern exhibits characteristic diffraction peaks at approximately 15°, 17°, 18° and 23°. As presented in Figure 8, all samples showed typical diffraction peaks at 15.3°, 17.3°, 18.1° and 23.3°, where the peaks at 17.3° and 18.1° formed a distinct doublet. This verified that Tartary buckwheat seed starch possessed a classic A-type crystalline structure. Furthermore, no new diffraction peaks appeared after ultrasound treatment, indicating that no new crystalline structures were generated. This finding is consistent with the results reported by Flores-Silva et al. [51] for ultrasound-treated corn starch.

The RC of starch was calculated from the diffraction peaks corresponding to crystalline and amorphous regions in X-ray diffractograms. The RC values of raw starch and ultrasound-assisted pre-germinated Tartary buckwheat starch subjected to different ultrasound durations were 14.35%, 14.41%, 15.78%, 17.42%, 16.80% and 16.60%, respectively. RC increased first and then decreased with prolonged ultrasound treatment, reaching a maximum at 60 min. Prior to 60 min of ultrasound treatment, the rise in RC could be ascribed to ultrasound-enhanced intermolecular forces between starch molecules, which promoted the formation of more ordered crystalline structures. Meanwhile, less stable crystal regions in the starch may be removed, and the remaining crystals underwent more complete re-crystallization [52]. However, when the ultrasound time was extended to 70 min, RC began to decline. This reduction was mainly caused by prolonged ultrasonic cavitation, which breaks intermolecular hydrogen bonds and destabilizes starch double-helical structures. Partial unwinding of the double helix induces local damage to crystalline regions, enlarges the amorphous proportion and improves molecular mobility.

#### 3.2.7. Effects of Ultrasound Treatment Duration on the FTIR Spectra of Starch in Tartary Buckwheat Seeds

**Figure 9:**
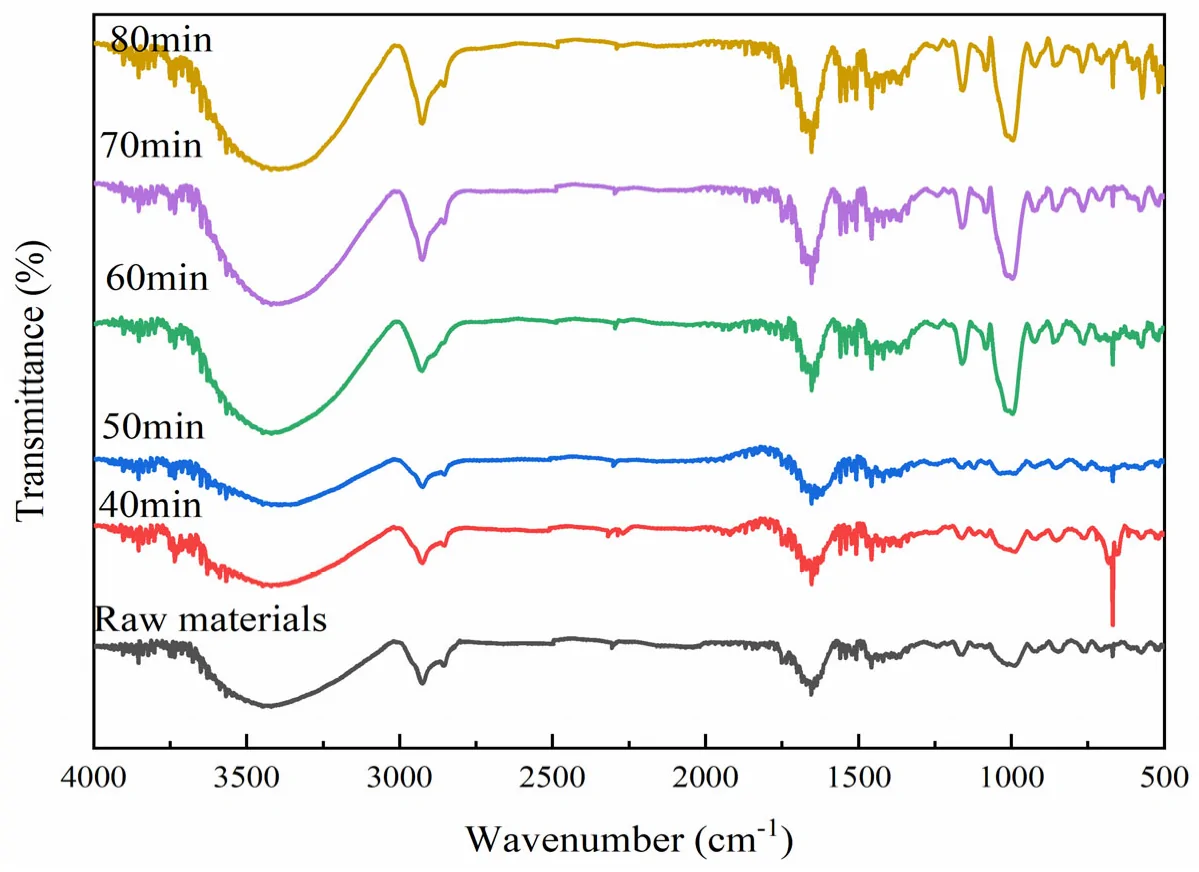
Infrared spectra of Tartary buckwheat starch with different ultrasonic time.

As shown in Figure 9, no new absorption bands appeared in the ultrasound-assisted pre-germinated Tartary buckwheat seeds, and the positions of existing peaks remained unchanged. This verified that ultrasound treatment only caused physical modifications to the starch, whereas its inherent chemical structure was not altered.

The FTIR spectra exhibited a broad absorption peak at approximately 3430 cm^−1^, which corresponds to O-H stretching vibrations associated with hydrogen bonding [53]. After 40–50 min ultrasound treatment, this peak was broad and blunt, indicating that outer amylopectin chains were cleaved into amylose and intermolecular hydrogen bonds were reduced. When the treatment time was extended to 60 min, the peak became narrower and sharper, suggesting increased intermolecular hydrogen bonding. Similarly, the absorption band near 1000 cm^−1^, assigned to C-OH bending vibrations related to hydrogen bonds [54], showed a relative increase in intensity after 60 min, confirming that extended treatment generates more amylose and exposes additional hydroxyl groups. The peak at 2927 cm^−1^, originating from C-H stretching vibrations reflecting the lipophilicity of starch [55], showed increased relative intensity at 60 min, indicating enhanced internal lipophilicity. Furthermore, the absorption at around 1649 cm^−1^, arising from O-H bending vibrations of bound water in starch amorphous regions [56], shifted from broad and weak at 40–50 min to relative narrow and strong at 60–80 min, demonstrating elevated water absorption capacity. Finally, the peak at 1370 cm^−1^ is attributed to C-H bending vibrations.

In FTIR spectroscopy, the absorbance ratios at 1047, 1022, and 995 cm^−1^ are commonly adopted to characterize alterations in the short-range molecular order of starch double helices. Specifically, the ratio of 1047/1022 cm^−1^ (R_1047/1022_) serves as a marker for short-range order degree, where the bands at 1047 cm^−1^ and 1022 cm^−1^ are attributed to the crystalline and amorphous regions, respectively. A decreased R_1047/1022_ value corresponds to reduced short-range ordering. Meanwhile, the ratio R_995/1022_ reflects the relative proportion of amorphous and ordered structures, representing the packing arrangement of double helices with starch crystallites. A lower R_995/1022_ indicates greater damage to the double-helical structure [57].

As presented in Table 6, prolonged ultrasound treatment caused a non-linear change in R_1047/1022_ value, which increased to a maximum at 50 min and exhibited no significant difference at 60 min. Meanwhile, the R_995/1022_ reached its peak (1.28 ± 0.01) after 60 min of ultrasound treatment. At 50 min, short starch chains preferentially undergo hydrogen-bond rearrangement to form abundant locally ordered segments, leading to an elevated degree of order (DO). Nevertheless, these ordered segments have not yet paired and become entangled into stable double helices. By 60 min, the locally ordered short chains further assemble and associate to form double helices; the ordered stacking of these double helices enlarges the crystalline domains, as reflected by the concurrent increase in the degree of double helix order (DD) and RC. When sonication is prolonged beyond 60 min, sustained cavitation shear disrupts the intermolecular hydrogen bonds of starch molecules, inducing double-helix unwinding and the breakdown of local ordered structures, which in turn causes concomitant reductions in DO, DD, and RC [58,59].

## 4. Conclusions

This study demonstrated that applying ultrasound-assisted pre-germination treatment following adequate water absorption significantly accelerates water uptake and increases the TFC in Tartary buckwheat seeds. Comparative analysis of TFC under different treatment regimes confirmed that the combined treatment consisting of water soaking followed by ultrasound-assisted germination was superior to direct ultrasound treatment before soaking and soaking-only in increasing TFC. The process parameters were optimized using single-factor experiments and response surface methodology (RSM). The optimal conditions were identified as 3 h of soaking, 60 min of ultrasound treatment and 20 h of pre-germination. Under these conditions, the contents of protein, fat, total starch, amylose and TFC were maximized. However, there were no significant differences in protein and fat contents between the 50 min and 60 min ultrasound treatments, nor were significant differences observed in amylose and resistant starch contents between the 60 min and 70 min ultrasound treatments. In raw Tartary buckwheat seeds without soaking, bound water was the predominant form, accompanied by semi-bound water. As ultrasound treatment duration and soaking time increased, bound water within Tartary buckwheat seeds migrated toward semi-bound water and free water. Furthermore, Ultrasound only influenced the granular morphology, intermolecular hydrogen bonds and RC of Tartary buckwheat seeds starch, whereas it exerted no significant effect on the degree of polymerization and crystal type. Current Tartary buckwheat processing is generally plagued by substantial losses of functional components such as flavonoids. Furthermore, conventional germination approaches suffer from sprout abscission and considerable raw material wastage. The optimal ultrasound-assisted germination protocol established herein (soaking for 3 h, ultrasound treatment for 60 min, and germination for 20 h) enables TFC enrichment in Tartary buckwheat seeds at a non-sprouting germinating stage, offering a viable processing strategy for developing high-quality functional Tartary buckwheat products. The present study did not evaluate single ultrasound or single pre-germination treatments of Tartary buckwheat seeds, cumulative temperature effects during ultrasonication and dynamic changes in enzyme activity over the pre-germination period; these aspects may be explored in future research.

## Figures and Tables

**Table 1:** Factor levels and coded values for response-surface methodology based on a three-factor three-level Box–Behnken design.

Level	A Soaking Time (h)	B Ultrasound Duration (min)	C Pre-Germination Duration (h)
−1	2	50	18
0	3	60	20
1	4	70	22

Note: All experimental runs were randomized.

**Table 2:** MRI analysis of internal water distribution in Tartary buckwheat seeds.

	Y1	Y2	Y3
1 h	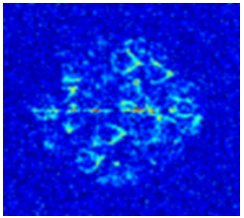	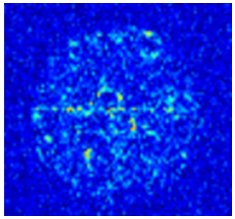	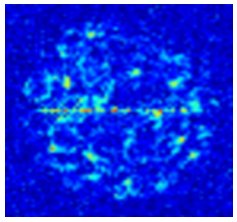
3 h	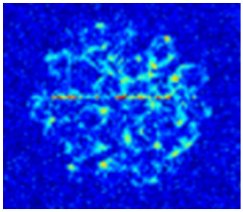	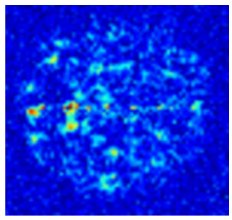	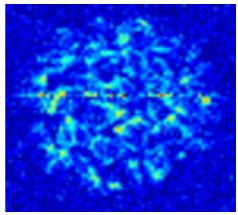
5 h	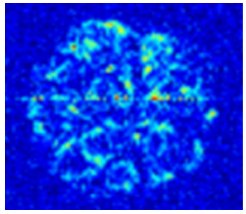	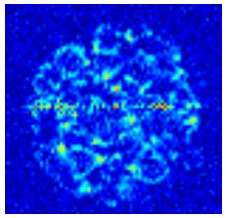	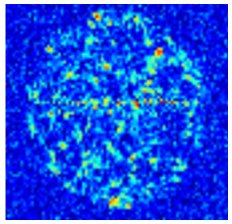
7 h	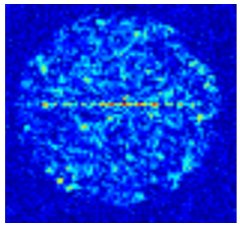	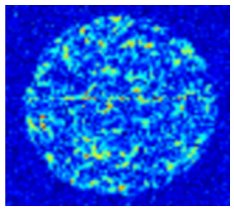	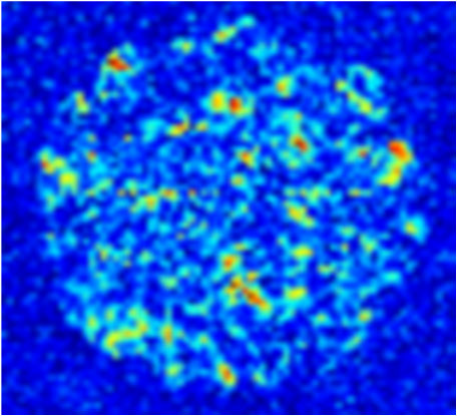

Y1: soaking 7 h; Y2: ultrasound 1 h + soaking 7 h; Y3: soaking 1 h + ultrasound 1 h + soaking 6 h.

**Table 3:** Response surface design and results of ultrasound-assisted pre-germination process.

Serial Number	A Soaking Time (h)	B Ultrasound Duration (min)	C Pre-Germination Duration (h)	TFC (mg/g)
1	4	60	18	23.94
2	3	60	20	24.48
3	4	60	22	22.98
4	4	70	20	22.84
5	3	50	22	24.05
6	3	60	20	24.39
7	4	50	20	23.58
8	3	50	18	23.66
9	2	50	20	23.39
10	3	60	20	24.42
11	3	60	20	24.37
12	2	60	18	23.00
13	3	60	20	24.49
14	3	70	22	22.85
15	3	70	18	23.56
16	2	60	22	23.94
17	2	70	20	23.26

**Table 4:** Response-surface analysis of variance results of ultrasound-assisted pre-germination process.

Source	Sum of Squares	df	Mean Square	*F*-Value	*p*-Value	Significance
Model	5.65	9	0.63	85.75	<0.0001	significant
A—Soaking time	7.81 × 10^−3^	1	7.81 × 10^−3^	1.07	0.3361	
B—Ultrasound time	0.59	1	0.59	80.36	<0.0001	***
C—pre-germination time	0.014	1	0.014	1.97	0.2029	
AB	0.093	1	0.093	12.7	0.0092	**
AC	0.9	1	0.9	123.21	<0.0001	***
BC	0.3	1	0.3	41.3	0.0004	**
A2	1.59	1	1.59	216.53	<0.0001	***
B2	1.27	1	1.27	173.09	<0.0001	***
C2	0.52	1	0.52	70.92	<0.0001	***
Residual	0.051	7	7.33 × 10^−3^			
Lack of Fit	0.04	3	0.013	4.66	0.0855	not significant
Pure Error	0.011	4	2.85 × 10^−3^			
Cor Total	5.7	16				

Note: ** and *** indicate the difference was significant, highly significant and extremely significant at 0.05, 0.01 and 0.001 level, respectively.

**Table 5:** Nutrients and flavonoids contents of Tartary buckwheat seeds with different ultrasonic duration.

Ultrasound Duration (min)	Protein (g/100 g)	Fat (g/100 g)	Total Starch (g/100 g)	Amylose (%)	Resistant Starch (g/100 g)	TFC (mg/g)
0 min (raw material)	11.81 ± 0.08 ^c^	2.31 ± 0.08 ^d^	58.76 ± 0.56 ^b^	22.45 ± 0.35 ^b^	4.82 ± 0.03 ^d^	20.17 ± 0.06 ^e^
40 min	12.47 ± 0.08 ^b^	3.00 ± 0.06 ^bc^	59.26 ± 0.02 ^b^	22.41 ± 0.69 ^b^	4.91 ± 0.01 ^c^	23.28 ± 0.07 ^cd^
50 min	12.72 ± 0.03 ^a^	3.28 ± 0.13 ^a^	58.62 ± 0.49 ^b^	22.19 ± 0.23 ^b^	4.88 ± 0.02 ^c^	23.48 ± 0.07 ^b^
60 min	12.74 ± 0.08 ^a^	3.33 ± 0.11 ^a^	61.61 ± 0.92 ^a^	24.86 ± 0.23 ^a^	5.37 ± 0.01 ^a^	24.35 ± 0.05 ^a^
70 min	12.71 ± 0.01 ^ab^	3.09 ± 0.14 ^ab^	59.12 ± 0.08 ^b^	24.37 ± 0.92 ^a^	5.41 ± 0.02 ^a^	23.37 ± 0.06 ^bc^
80 min	12.54 ± 0.18 ^ab^	2.84 ± 0.05 ^c^	56.57 ± 0.09 ^c^	23.13 ± 1.16 ^ab^	5.03 ± 0.02 ^b^	23.18 ± 0.12 ^d^

Note: Mean differences were assessed by Duncan’s multiple range test. Data are presented as mean ± standard deviation (*n* = 3). Different letters show significant variations (*p* < 0.05) between values in the same column.

**Table 6:** Short-range ordered structure of Tartary buckwheat seeds starch at different ultrasonic time.

Treatment	R_1047/1022_	R_995/1022_
0 min (raw material)	0.75 ± 0.04 ^c^	1.08 ± 0.02 ^b^
40 min	0.81 ± 0.01 ^b^	1.05 ± 0.04 ^b^
50 min	0.88 ± 0.01 ^a^	1.02 ± 0.04 ^b^
60 min	0.86 ± 0.02 ^ab^	1.28 ± 0.01 ^a^
70 min	0.69 ± 0.01 ^d^	1.05 ± 0.01 ^b^
80 min	0.68 ± 0.01 ^d^	1.07 ± 0.01 ^b^

Note: Values followed by different lowercase letters within the same column are significantly different at *p* < 0.05.

## Data Availability

The original contributions presented in the study are included in the article; further inquiries can be directed to the corresponding author.
